# Association of childbirth experience with long–term psychological outcomes: a prospective cohort study

**DOI:** 10.1186/s12978-024-01819-9

**Published:** 2024-05-30

**Authors:** Jila Nahaee, Mansour Rezaie, Elham Abdoli, Mojgan Mirghafourvand, Solmaz Ghanbari-Homaie, Mina Jafarzadeh

**Affiliations:** 1 Department of Midwifery, Faculty of Nursing and Midwifery, Tehran Medical Sciences, Islamic Azad University, Tehran, Iran; 2 Department of Anesthesiology, School of Medicine, Tabriz University of Medical Sciences, Tabriz, Iran; 3 Student Research Committee, Tabriz University of Medical Sciences, Tabriz, Iran; 4 Social Determinants of Health Research Center, Tabriz University of Medical sciences, Tabriz, Iran; 5 Department of Midwifery, Faculty of Nursing and Midwifery, Tabriz University of Medical Sciences, Tabriz, Iran

**Keywords:** Birth satisfaction, Mental health, Sexual health, Reproduction

## Abstract

**Background:**

There has been limited research on the lasting impact of giving birth on both mothers and infants. This study aimed to investigate women’s memories of their childbirth experience 4 months and 4 years after giving birth. Additionally, it aimed to examine how the childbirth experience is linked to women’s mental health, sexual satisfaction, exclusive breastfeeding, and the type of subsequent birth.

**Methods:**

In this prospective cohort study, a total of 580 women giving birth in Tabriz hospitals in 2018 were followed up for 4 years. The data were collected using a childbirth experience questionnaire, a mental health inventory, and a sexual satisfaction scale for women, and were analyzed by a Pearson correlation test, an independent samples t-test, and a general linear model.

**Results:**

The total scores of the childbirth experience in two short-term (4 months) and long-term (4 years) time points following the birth had a significant and strong correlation with each other (*r* = .51; *p* < .001). After adjusting for the effects of socio-demographic and obstetric characteristics, sexual satisfaction had significant relationships with childbirth experience (*p* < .001) and postpartum complications (*p* < .001). In addition, mental health had significant relationships with childbirth experience (*p* < .001), postpartum complications (*p* < .001), and low income (*p* = .004).

**Conclusions:**

Even 4 years after giving birth, women have a clear recall of their childbirth experience. This experience has a significant association with long-term outcomes such as sexual satisfaction, mental health, exclusive breastfeeding, and subsequent birth type.

## Plain Language Summary

Childbearing is a significant event in a mother’s life. Women often remember the births of their babies distinctly, even after many years. However, some mothers do not perceive this life event as a positive one. Factors affecting this perception are multidimensional and may include demographic variables and obstetric events (e.g., immediate postpartum complications like hemorrhage), which contribute to negative childbirth experiences. It’s important to note that women who feel unsupported or lack control during labour and childbirth are more likely to have negative birth experiences. Although a birth experience is influenced by various factors, this can have serious consequences, such as mental health problems, disrupted mother-infant bonding, and reluctance to have subsequent pregnancies or initiate or continue breastfeeding. Therefore, this study mainly aimed to investigate women’s recollection of their childbirth experience at 4 months and 4 years after giving birth, as well as examine the correlation of childbirth experience with women’s mental health, sexual satisfaction, exclusive breastfeeding, inclination to have children, and the type of next birth 4 years after the childbirth.

## Background

Childbirth is a profound and transformative event in a woman’s life that has a significant impact on her physical, emotional, and mental well-being [1]. Although all mothers experience some initial biological changes, each mother has a unique perception of the experience [2]. The experience of childbirth is a complex event that is influenced by multiple factors [3–5]. After giving birth, women from various cultures can vividly recall their experiences, even after an extended period of time. This phenomenon greatly shapes a woman’s perspective on childbirth [6–8].

Childbirth experience can have a significant impact on a woman’s personal and family life [9]. Experiencing negative childbirth can have both short- and long-term effects on women’s mental health. A negative childbirth experience may cause postpartum depression [10], post-traumatic stress disorder (PTSD) [9, 11], maternal mental health [12], sexual dysfunction [9], as well as influence breastfeeding [13], childbearing tendency, and the type of subsequent birth [14].

 In a study carried out in England, women experiencing a traumatic vaginal birth had higher levels of sexual dissatisfaction and dyspareunia than those having an elective cesarean Sect. [15]. Negative childbirth experiences can have negative impacts on a woman’s mental health and relationships with her family [16]. According to the literature, the childbirth experience is significantly associated with women’s short-term postpartum mental health and their high levels of postpartum irritability [17, 18]. The stress resulting from a negative childbirth experience can affect a woman’s mental health and disrupt the mother-infant bonding [19, 20].

One factor that may contribute to women’s reluctance to have subsequent pregnancies is their previous negative childbirth experience [21–23]. A study on primiparous women found that those who had negative childbirth experiences were more likely to have C-sections in subsequent pregnancies [24]. Fear of childbirth is the primary reason why women opt for cesarean delivery for subsequent pregnancies over vaginal birth [25, 26]. A negative childbirth experience may discourage breastfeeding initiation or continuation in the short term and encourage the use of infant formula in the long term [2]. On the other hand, mothers who have a positive childbirth experience tend to breastfeed their infants for longer than 9 months [27].

According to the literature, several researchers have investigated the effects of childbirth experience on some maternal and neonatal outcomes in the short-term period [28–30]. To date, however, there has been little research on these factors over a long-term period [6, 8]. In addition, certain outcomes, such as sexual satisfaction, breastfeeding duration, child-bearing tendency, and subsequent birth type, have not been adequately researched over long periods of time. Also, childbirth experience has assessed using one item with limited options (happy or unhappy).

Due to the high prevalence of negative childbirth in Iran (38%) [31], it is important to investigate the effects of childbirth experience on maternal mental health and other relevant variables. Therefore, the current study mainly aimed to investigate women’s recollection of their childbirth experience at4 months and 4 years after the births of their babies (primary aim). It also aimed to examine the correlation of the childbirth experience with women’s mental health, sexual satisfaction, exclusive breastfeeding, childbearing tendency, and the type of subsequent birth at 4 years postpartum (secondary aims).

## Methods

### Study Design and participants

In this prospective cohort study, a total of 580 primiparous women giving birth in Tabriz hospitals, Iran, in 2018 [31] were followed up for 4 years. The sampling criteria were the subjects’ willingness to participate in the study and their willingness to have telephone interviews with the researcher. Those unwilling to have telephone structured interviews with the researcher (due to migration) and those unable to complete at least 80% of the questionnaires were excluded from the study.

## Sample size

A total of 800 women (580 women from the urban areas of Tabriz and 220 women from its suburban areas) were included in the previous study [31]. In this study, only women from the urban areas were followed up. The post-hoc power of the study was calculated using G*Power software and it was estimated approximately 90%.

### Procedure

In the previous study, Ghanbari et al. examined the childbirth experience of 800 women between 1 and 4 months postpartum [31]. Out of 800 women, those regularly attending urban health centers were included in the present study, and those unavailable for the researcher due to different reasons (e.g., those who migrated to other places) were excluded. The eligible women were given phone calls by the researcher and briefed on the study objectives and procedure. Therefore, social-demographic information and birth characteristics, the Childbirth Experience Questionnaire version 2.0 (CEQ 2.0), the Mental Health Inventory (MHI), the Sexual Satisfaction Scale for Women (SSS-W), and the maternal and neonatal outcomes checklist were completed for the interviewees on the phone. It should be noted that the participants giving birth to another child were requested to complete the CEQ 2.0 based on the information about their second birth. The completed questionnaires were coded to protect the participants’ privacy and confidentiality.

### Data collection tools

The data were collected using the CEQ, MHI, and SSS-W (the CEQ was completed 4 months and 4 years after the birth). The maternal and neonatal outcome checklist was also used for data collection.

#### Mental Health Inventory (MHI)

The short form of MHI was used to collect the relevant data on the participants’ mental health. This 18-item tool had four domains, including anxiety, depression, behavioral control, and positive affect. The items were scored on a six-point scale including, *always* (score 1), *often* (score 2), *most of the time* (score 3), *sometimes* (score 4), *rarely* (score 5), and *never* (score 6). Reverse scoring was used for items 5, 3, 1, 7, 8, 10, 13, and 15. The total scores ranged from 18 to 108, with higher scores indicating a better mental health status [32]. The validity and reliability of this scale have been approved by Meybodi et al. (2011), who assessed the psychometric properties of MHI in Iran. Cronbach’s alpha coefficient for this scale was reported as 0.93 [32, 33].

#### Childbirth experience Questionnaire Version 2.0 (CEQ 2.0)

This 23-item questionnaire was used to assess the childbirth experiences of the participants. The CEQ had four domains, including personal capacity, professional support, perceived security, and participation. It consisted of 20 four-option items and three other items that were scored using a visual analogue scale (VAS). The responses included *totally agree* (score 4), *mostly agree* (score 3), *mostly disagree* (score 2), and *totally disagree* (score 1). The visual analogue scale values were converted to scores from 1 to 4: 0–40 (score 1), 41–60 (score 2), 61–80 (score 3), and 80–100 (score 4). Reverse scoring was used for items 3, 5, 8, 13, 14, 19, 20, and 21. The total scores ranged from 1 to 4, with higher scores indicating a more positive childbirth experience [34]. Furthermore, the total scores ≤ 2.50 indicated a negative experience. Ghanbari et al. assessed the psychometric properties of the CEQ in Iran [35]. Also, Cronbach’s alpha coefficient of the CEQ 2.0 was calculated in the current study as 0.90.

#### Sexual satisfaction scale for women (SSS-W)

This 30-item scale included five domains of contentment, communication, compatibility, relational concern, and personal concern. The items were scored on a five-point scale, including *completely agree* (score 1), *slightly agree* (score 2), *neither agree nor disagree* (score 3), *slightly disagree* (score 4), and *completely disagree* (score 5). Reverse scoring was used for items 1, 4, 5, 6, 9, 10, 11, and 12. The total scores ranged from 30 to 150, and higher scores indicated higher levels of sexual satisfaction. Roshan Chesli et al. (2014) assessed the psychometric properties of this tool in Iran [36].

#### Maternal and neonatal outcome checklist

This checklist included questions such as the second birth, exclusive breastfeeding, duration of breastfeeding, type of subsequent birth, reasons for women’s reluctance to have a second child, and postpartum complications.

## Ethical considerations

The study was approved by the Ethics Committee of Tabriz University of Medical Sciences (ethical code: IR.TBZMED.REC.1400.1222), and an informed consent was obtained from all the participants using the online form. Moreover, the study was conducted in accordance with the Helsinki Declaration.

### Data analysis

The data were analyzed using the SPSS software (version 24) for Windows (IBM Inc., Armonk, NY, USA). Descriptive statistics, including frequency (percentage) and mean (standard deviation), were used to describe the qualitative and quantitative data, respectively. A Pearson correlation test was performed to examine the relationship between the primiparous women’s childbirth experience at 4 months and 4 years postpartum. Those women who had their second birth or were pregnant were excluded from the analysis. In addition, another Pearson correlation test was performed to examine the relationship between the mothers’ childbirth experience and their mental health or sexual satisfaction at 4 years postpartum. The relationships of the mothers’ childbirth experience with exclusive breastfeeding, subsequent pregnancy, and the type of subsequent birth at 4 years postpartum were assessed using an independent samples t-test. In the next step, the significant variables (*p* < .1) were entered into the general linear model, and the correlations of childbirth experience with mental health and sexual satisfaction were assessed after adjusting the effect of these variables. All General Linear Model assumptions (linearity, constant variance, normality, and independence) were tested and confirmed prior to performing this test. Finally, *p*-values < 0.05 were considered significant.

## Results

Out of all eligible women from the urban areas of Tabriz (*n* = 580), 462 women were enrolled in the study (response rate = 79.6%) from May to December, 2022. The women who failed to answer the phone calls to their registered numbers or unwilling to participate in the study were excluded.

The mean age of the participants was 24 ± 4.8 years. In total, 445 women (96.9%) breastfed their infants, and the mean duration of the breastfeeding was 21 ± 5.6 months. Furthermore, 85 women (18.4%) had given birth to their second babies, out of whom 66 women (77.6%) had had vaginal birth. Previous negative childbirth experience (10.6%), financial problems (19%), family problems (96.9%), intentional spacing between pregnancies (11%), and other reasons (i.e., secondary infertility, divorce, illness, difficult job situation, and death of spouse) (5.2%) were the main factors responsible for women’s unwillingness to have more children. In the present study, about 2.8% of the women were pregnant during the study. Out of all the pregnant women, nearly 85% reported that their pregnancies were wanted, and 77% intended to have vaginal birth (Table 1).

**Table 1:** Socio-demographic and birth characteristics (*n* = 462)

Variables	*n* (%)
Age, Mean (SD)	24.0 (4.8)
Educational level	
Illiterate or elementary	50 (10.8)
Secondary or high school	155 (33.6)
Diploma	170 (36.8)
Academic	87 (18.8)
Income adequacy	
Inadequate	88 (19.0)
Relatively adequate	323 (69.9)
Completely adequate	51 (11.0)
Insurance	274 (59.3)
Unwanted pregnancy	49 (10.6)
Labour augmentation	314 (68.0)
Pain relief during labour	190 (41.1)
Companion during labour	83 (18.0)
Episiotomy	453 (98.0)
Skin-to skin-contact	404 (87.4)
Breastfeeding within an hour of birth	278 (60.2)
Breastfeeding	445 (96.9)
Breastfeeding duration (Month), Mean (SD)	20.9 (5.6)
Postpartum complications	57 (12.3)
History of abortion after first birth	35 (7.7)
Second birth	85 (18.4)
Wanted second pregnancy	54 (62.8)
Type of second birth, vaginal	66 (77.6)
Reasons for being unwilling with second reproduction	
Pervious negative childbirth experience	26 (7.1)
Financial issues	139 (38.2)
Familial issues	141 (38.7)
Tendency for spacing between pregnancies	40 (11.0)
Other reason^a^	18 (5.0)
Current pregnancy	13 (2.8)
Wanted current pregnancy	11 (84.6)
Preferred birth type, vaginal	10 (76.9)

^a^Tendency to have just one child; Difficult job conditions; Medical problems; Secondary infertility; Divorce; Husband Death

### Association of childbirth experience and its subscale scores at 4 months and 4 years following birth

The total scores of the childbirth experience in two short-term (4 months) and long-term (4 years) time points following the birth had a significant and strong correlation with each other (*r* = .51; *p* < .001). Also, three childbirth experience’s subscales including own capacity (*r* = .38; *p* < .001), perceived safety (*r* = .37; *p* < .001), and participation (*r* = .35; *p* < .001) in two short-term and long-term time points had a significant and moderate correlation with each other among primiparous women (Table 2).

**Table 2:** Association between Childbirth experience and its subscales scores at four months and four years after birth

Variables	Four Months *n* = 462	Four Years^*^ n = 364	*r* (p^**^)
	Mean (SD)		
Total score (1 to 4)	2.7 (0.7)	2.9 (0.7)	0.51 (< 0.001)
Own capacity (1 to 4)	2.6 (0.8)	2.6 (0.7)	0.38 (< 0.001)
Participation (1 to 4)	2.7 (0.8)	3.0 (0.9)	0.35 (< 0.001)
Perceived safety (1 to 4)	2.6 (0.8)	2.9 (1.2)	0.37 (< 0.001)
Professional support (1 to 4)	2.8 (0.9)	2.5 (0.4)	0.05 (0.340)
Negative childbirth experience, n (%)	128 (35.2)	111 (30.4)	

*Women who had second birth or pregnant were excluded from analysis; **Pearson Correlation Test

### Association of childbirth experience with sexual satisfaction and mental health 4 years following birth

The childbirth experience was significantly and moderately correlated with the total scores for sexual satisfaction score (*r* = .31; *p* < .001) and its subscales: contentment (*r* = .25; *p* < .001), communication (*r* = .24; *p* < .001), compatibility (*r* = .20; *p* < .001), relational concern (*r* = .29; *p* < .001), and personal concern (*r* = .23; *p* = .028) (Table 3). Moreover, the childbirth experience was weakly significantly correlated with the total scores for MHI (*r* = .17; *p* < .001) and its subscales: anxiety (*r* = .10; *p* = .040), depression (*r* = .14; *p* < .001), behavioral control (*r* = .13; *p* = .013), and positive affect (*r* = .23; *p* < .001) (Table 3).

**Table 3:** Correlation of childbirth experience with women’s sexual satisfaction and mental health four years after birth (*n* = 364)

Variables	Mean (SD)	Childbirth Experience
		*r* (*p*)^*^
SSSW^**^	140.7 (17.2)	0.31 (< 0.001)
Contentment	27.2 (4.7)	0.25 (< 0.001)
Communication	27.8 (4.1)	0.24 (< 0.001)
Compatibility	28.4 (5.0)	0.20 (< 0.001)
Concern Relational	28.5 (3.4)	0.29 (< 0.001)
Concern Personal	28.6 (5.2)	0.23 (0.028)
MHI^***^	82.3 (14.3)	0.17 (0.001)
Anxiety	24.1 (5.1)	0.10 (0.040)
Depression	19.4 (4.5)	0.14 (< 0.001)
Behavioral control	17.3 (4.2)	0.13 (0.013)
Positive affect	18.4 (4.0)	0.23 (< 0.001)

^*^Pearson Correlation test; ^**^SSSW = Sexual Satisfaction Scale for Women; ^***^MHI = Mental Health Inventory

### The relationship of childbirth experience and some maternal and neonatal outcomes 4 years following birth

The consequences of the subsequent type of birth and breastfeeding have been investigated in only 85 women. The childbirth experience was significantly associated with the type of subsequent birth (*p* = .003) and exclusive breastfeeding (*p* = .003). However, there was no significant association between childbirth experience and subsequent childbearing tendency (*p* = .950) (Table 4).

**Table 4:** Correlation of childbirth experience with some maternal and neonatal outcomes four years after birth (*n*= 462)

Variable	Childbirth Experience	P^*^
Childbearing		0.950
Yes	2.7 (0.7)	
No	2.7 (0.7)	
Type of second birth (*n* = 85)		**0.003**
CS	2.3 (0.7)	
Vaginal	2.8 (0.6)	
Breastfeeding		**0.003**
Yes	2.7 (0.7)	
No	2.2 (0.6)	
Wanted second pregnancy		0.076
Yes	3.1 (0.6)	
No	2.2 (0.03)	
History of abortion after first birth		0.931
Yes	2.7 (0.7)	
No	2.7 (0.7)	

^*^Independent T-test

### Association between socio-demographic and birth characteristics with sexual satisfaction and mental health

Out of the socio-demographic and obstetric characteristics of the participants, sexual satisfaction had a significant relationship with postpartum complications (*p* = .006), while mental health had significant relationships with mean age (*p* = .028), low income (*p* < .001), and postpartum complications (*p* < .001) (Table 5). After adjusting for the effects of socio-demographic and obstetric characteristics, sexual satisfaction was found to have significant relationships with childbirth experience (*p* < .001) and postpartum complications (*p* < .001). In addition, mental health had significant relationships with childbirth experience (*p* < .001), postpartum complications (*p* < .001), and low income (*p* = .004) (Table 6).

**Table 5:** Correlation of socio-demographic and birth characteristics with women’s sexual satisfaction and mental health four years after Birth (*n*= 364)

Variable	Sexual Satisfaction		Mental Health	
	Mean (SD)	P^*^	Mean (SD)	P^*^
Age, Mean (SD)	-0.05^**^	0.223	-0.10^**^	**0.028**
Educational level		0.757		0.477
Illiterate or elementary	140.7 (20.4)		79.3 (17.2)	
Secondary or high school	138.9 (18.6)		82.5 (13.6)	
Diploma	140.8 (17.7)		81.7 (13.9)	
Academic	141.0 (17.6)		83.1 (15.1)	
Income adequacy		0.128		**< 0.001**
Inadequate	137.5 (21.5)		76.8 (15.5)	
Relatively adequate	140.3 (18.1)		82.6 (14.0)	
Completely adequate	144.0 (11.9)		86.8 (12.5)	
Wanted pregnancy		0.186		0.214
Yes	140.9 (17.7)		82.6 (13.9)	
No	136.3 (22.6)		80.0 (16.4)	
Postpartum complication		**0.006**		**< 0.001**
Yes	131.1 (26.5)		73.0 (17.9)	
No	141.5 (16.4)		83.2 (13.4)	
Episiotomy		0.557		-
Yes	140.1 (18.4)			
No	143.7 (9.5)			

^*^Analyses were performed by Pearson Correlation test; One-Way ANOVA; and Independent T-test; ^**^r

**Table 6:** Correlation of childbirth experience with women’s sexual satisfaction and mental health four years after birth (*n*= 364)

Variable	Sexual Satisfaction		Mental Health	
	B (95% CI)	P^*^	B (95% CI)	P^*^
Childbirth Experience	4.8 (2.5 to 7.0)	**< 0.001**	3.2 (1.4 to 5.0)	**< 0.001**
Postpartum complication (Reference: Yes)		**< 0.001**		**< 0.001**
No	9.5 (4.6 to 14.5)		8.9 (5.1 to 12.7)	
Age, Mean (SD)	-		-0.18 (-0.44 to 0.07)	0.165
Income adequacy (Ref: Completely adequate)	-			
Inadequate			-6.12 (-11.9 to -2.2)	**0.004**
Relatively adequate			-2.34 (-6.6 to 1.54)	0.222
R^2^	0.690		0.118	

^*^General Linear Model

## Discussion

This study mainly aimed to investigate women’s recollection of their childbirth experience at 4 months and 4 years after the birth of their babies. It also aimed to investigate the correlation of the childbirth experience with their mental health, sexual satisfaction, exclusive breastfeeding, and the type of subsequent birth.

A significant association was found between the CEQ scores in two periods of time, namely the short-term (4 months) and long-term (4 years) postpartum periods in primiparous women. Childbirth experience was significantly associated with the type of subsequent birth and breastfeeding; however, it had no significant relationship with subsequent childbearing. In addition, childbirth experience and postpartum complications had significant relationships with sexual satisfaction. Finally, childbirth experience, postpartum complications, and low income were significantly associated with the mental health.

There was a significant association between the CEQ scores of the primiparous women at 4 months postpartum and their CEQ score at 4 years postpartum. This result is consistent with the findings of a study carried out in the Netherlands [6], showing that 94% of the women clearly remembered their childbirth experiences at 3 years postpartum. Labour complications such as operative vaginal birth and unplanned (emergency) C-section increased the risk of negative recall of the previous childbirth experiences [6]. A study in Japan used the Childbirth Experience Scale (CBE-Scale) to assess the childbirth experiences of 1168 women a few days after giving birth and to follow up on the childbirth experiences of 584 other women for 5 years. It was found that women still remembered their childbirth experiences clearly 5 years after the childbirth [37]. Contrary to the findings of the present research, the results of a study in Turkey showed that women had only moderate recall of their childbirth experience at 1 year postpartum [38]. This inconsistency may be due to the fact that the Turkish study used the Birth Memories and Recall Questionnaire, which is a relatively low-grade tool compared to the CBE-Scale for measuring childbirth experiences [39].

The childbirth experience was significantly associated with the subsequent birth types (C-section or vaginal) and breastfeeding; however, it had no significant relationship with the subsequent birth. A systematic review examined 12 studies, out of which five articles found a positive association between women’s negative childbirth experience and their unwillingness to have more children; three studies found a positive association between women’s negative childbirth experience and their willingness to postpone their next pregnancy; and six studies showed a positive association between women’s negative childbirth experience and their request for a C-section in their subsequent pregnancies. Most of the studies included in the systematic review used only one question (rather than a standard tool) to assess childbirth experience. In some studies, postpartum complications were also considered causes of the negative birth experience. This may be one of the factors contributing to the discrepancy between our study results and those from the systematic review [40].

In the previous study conducted on the same sample, childbirth experience was found to have a significant relationship with childbearing tendency and subsequent vaginal birth [28]. The conflicting results may be attributed to the different durations of postpartum follow-up periods in these studies. In the previous study, childbearing tendency was measured in the first few months (1–4 months) postpartum; thus, the responses may not have been accurate due to the particular postpartum conditions and the lack of peace of mind. In line with our study results, the findings from a study in Sweden demonstrated that the type of birth and negative childbirth experience did not influence primiparous women’s childbearing tendency at 9 months and 5 years postpartum [41].

The childbirth experience had a significant relationship with exclusive breastfeeding. Traumatic childbirth experiences can result in poor breastfeeding, other nutritional problems, and delayed mother-infant bonding (due to the mother’s negative perception of the breastfeeding infant) [42]. After adjusting for the social factors (e.g., breastfeeding shame and perceived support), mothers with negative experiences were found to stop breastfeeding more often than others because of the pain and other breastfeeding problems [13].

Childbirth experience and postpartum complications had significant associations with sexual satisfaction. In a study in England [9], childbirth-related PTSD disrupted the marital relationships of the couples. Women experienced sexual dissatisfaction since they were afraid of getting pregnant and going through the same negative experience, which is consistent with our study results. The reported prevalence of sexual disorders in the postpartum period varies from 22 to 86% [43]. Women and men experience high levels of sexual dissatisfaction due to the decreased postpartum libido [44]. Delayed resumption of postpartum sexual intercourse or postpartum sexual abstinence and disrupted relationships between a wife and her husband and family are among the negative consequences of a negative childbirth experience [42]. Men may also be affected by the childbirth experience of their wives. A study has suggested that unexpected events during pregnancy and childbirth can trigger “extreme excitement” in men. Postpartum complications and problems are a series of events that directly or indirectly influence the sexual relationship between the spouses after childbirth. These problems are even more complex for mothers with traumatic childbirth experiences [45].

Childbirth experience, postpartum complications, and low-income status were significantly associated with postpartum mental health. In a prospective longitudinal study in Turkey, some pregnant women with a gestational age of 36–40 weeks were followed up until 6 weeks after the birth, and it was discovered that about 21% of them had traumatic childbirth experiences associated significantly with postpartum depression, anxiety, and stress [46]. Consistent with our study results, the findings of a cross-sectional study carried out in Iran on 483 mothers at 4–16 weeks postpartum showed a significant direct relationship between the participants’ childbirth experience and their mental health status [18]. In addition, women with low-income status were more likely to display high-risk behaviors and less likely to seek social support when feeling distressed; therefore, this group of women was more vulnerable to the deterioration of their mental state [47].

### Strength and limitations

This study has a few limitations. Firstly, our researcher was unable to contact women from the suburbs of Tabriz to carry out the necessary follow-ups. Secondly, the participants’ baseline mental health and sexual satisfaction scores were not evaluated in this study. However, the study’s greatest strength lies in the fact that it successfully recorded a high response rate of participants from the urban areas of Tabriz (79.6%) and followed up on the long-term outcomes of their childbirth experience. Most previous studies have assessed the childbirth experience with one Likert question. While, in this study, it was evaluated at both time points using standard scale. Also, the consequences of the subsequent type of birth and breastfeeding have been investigated in only 85 women; therefore, according to this number of samples (85 women), it is not possible to consider the final result about this outcome for all samples. Completing the questionnaire by phone interview in this study can be another limitation; however, it was tried that all the questionnaires were completed in the same way and by one researcher in order to prevent bias as much as possible.

## Conclusion

Women have a remarkable memory of their childbirth experience (measuring using standard tool not one single question), even after four years of giving birth. It also emphasizes the importance of childbirth experience and its relationship with long-term and important consequences such as mental health status, sexual satisfaction, exclusive breastfeeding, and the type of subsequent birth. Policymakers are recommended to focus on the childbirth process and implement effective and evidence based strategies to improve women’s childbirth experience, which in turn will improve postpartum psychological and physical outcomes. Also, healthcare professionals should provide more support during the postpartum period to women who had a negative experience of childbirth. It is suggested to investigate other psychological outcomes for a longer period in future studies. Also, childbirth experience and its relationship with postpartum outcomes in women who underwent cesarean section also should be explored.

## Data Availability

The datasets used and analyzed during the current study are available to be delivered by the corresponding author on reasonable request.
